# Early Retinal Defects in *Fmr1*^−/y^ Mice: Toward a Critical Role of Visual Dys-Sensitivity in the Fragile X Syndrome Phenotype?

**DOI:** 10.3389/fncel.2018.00096

**Published:** 2018-04-06

**Authors:** Olivier Perche, Chloé Felgerolle, Maryvonne Ardourel, Audrey Bazinet, Arnaud Pâris, Rafaëlle Rossignol, Géraldine Meyer-Dilhet, Anne-Laure Mausset-Bonnefont, Betty Hébert, David Laurenceau, Céline Montécot-Dubourg, Arnaud Menuet, Jean-Charles Bizot, Jacques Pichon, Isabelle Ranchon-Cole, Sylvain Briault

**Affiliations:** ^1^Genetic Department, Centre Hospitalier Régional d’Orléans, Orléans, France; ^2^UMR7355, Immunologie et Neurogénétique Expérimentales et Moléculaires (INEM), Centre National de la Recherche Scientifique, Orléans, France; ^3^Experimental and Molecular Immunology and Neurogenetics, University of Orléans, Orléans, France; ^4^IRMB, University of Montpellier, INSERM, Montpellier, France; ^5^KeyObs, CRO Pharmacology, Orléans, France; ^6^Laboratory of Sensorial Biophysical, INSERM UMR1107 Equipe Biophysique Neurosensorielle, University of Clermont 1, Clermont-Ferrand, France

**Keywords:** Fragile X syndrome, Fmrp, vision, sensorial dys-sensitivity, peripheral nervous system

## Abstract

Fragile X Syndrome (FXS) is caused by a deficiency in Fragile X Mental Retardation Protein (FMRP) leading to global sensorial abnormalities, among which visual defects represent a critical part. These visual defects are associated with cerebral neuron immaturity especially in the primary visual cortex. However, we recently demonstrated that retinas of adult *Fmr1*^−/y^ mice, the FXS murine model, present molecular, cellular and functional alterations. However, no data are currently available on the evolution pattern of such defects. As retinal stimulation through Eye Opening (EO) is a crucial signal for the cerebral visual system maturation, we questioned the precocity of molecular and functional retinal phenotype. To answer this question, we studied the retinal molecular phenotype of *Fmr1*^−/y^ mice before EO until adult age and the consequences of the retinal loss of Fmrp on retinal function in young and adult mice. We showed that retinal molecular defects are present before EO and remain stable at adult age, leading to electrophysiological impairments without any underlying structural changes. We underlined that loss of Fmrp leads to a wide range of defects in the retina, settled even before EO. Our work demonstrates a critical role of the sensorial dysfunction in the *Fmr1*^−/y^ mice overall phenotype, and provides evidence that altered peripheral perception is a component of the sensory processing defect in FXS conditions.

## Introduction

Loss of Fragile X Mental Retardation Protein (FMRP) induces alterations of neuronal synapses either in their structure or in their functions (Irwin et al., 2000; Nimchinsky et al., 2001) and thus leads to the human condition known as the Fragile X Syndrome (FXS). This neuropsychiatric pathology is the most common form of inherited intellectual and behavioral disabilities associated with hypersensitivity to sensory stimuli (Penagarikano et al., 2007; Hagerman and Hagerman, 2015). Indeed, the direct clinical observation of children with FXS led clinicians to suggest the existence of sensorial disturbance. Early in life these children have a strong aversion for tactile contact, an increased sensitivity to noises and an over-sensitivity to light or face recognition defect (Wolff et al., 1989; Lachiewicz et al., 1994; Merenstein et al., 1996).

Concerning vision, it has been shown that visual signal integration is particularly affected in FXS patients, with alteration of spatiotemporal visual processing, reduction of contrast sensitivity for visual stimuli presented at high temporal frequencies, and visual sensitivity for both static (texture difference) and moving images (Kogan et al., 2008; Farzin et al., 2011). These visual defects are associated with cerebral neuron immaturity (Irwin et al., 2000; Bilousova et al., 2009) especially in the primary visual cortex (Berman et al., 2012). However, we recently demonstrated that, in mice, Fmrp is also expressed in the retina, the visual nervous peripheral system (Rossignol et al., 2014), which is the first structure responsible for light transduction. Moreover, in collaboration with Guimarães-Souza et al. (2016) we showed that the Fmrp retinal content is regulated by light exposure. In the same way, investigations on drosophila’s retinas demonstrated a key role of Fmrp in Rhodopsin regulation (Wang et al., 2017). As Rhodopsin is the light sensor of photoreceptor cells, these studies highlighted the leading role of Fmrp in retinal function. All these reports are in line with our previous investigation demonstrating the retinal impact of Fmrp absence in the *Fmr1*^−/y^ mice (Rossignol et al., 2014), the murine model of FXS (Bakker et al., 1994). In 6 month-old mice, we demonstrated significant abnormalities in the signal transmission between photoreceptors and the inner retina, measured by electroretinogram (ERG) technique, associated to protein defects, such as Rhodopsin and PSD95, and cellular alterations in the retina (Rossignol et al., 2014). Therefore absence of Fmrp seems to lead to an overall visual defect starting from the perception of light by the neural retina to cerebral visual areas as showed in FXS patients (Kogan et al., 2008; Farzin et al., 2011). However, no data are currently available on the chronological evolution pattern of such defects. This is even more interesting since, under physiological conditions, the starting point of cerebral visual system maturation is the retinal light sensoring by Rhodopsin occurring during Eye Opening (EO; Gandhi et al., 2008), which ends up in a massive synaptogenesis in the primary visual cortex (Blue and Parnavelas, 1983; Gandhi et al., 2005). We could therefore hypothesize that any retinal alterations occurring before EO should lead to visual cortical immaturity. Since adult *Fmr1*^−/y^ retinas present alterations, we investigated the chronological order of this defect especially before EO by exploring molecular, structural and functional features before EO and in young and adult *Fmr1*^−/y^ retinas.

We highlighted for the first time that in *Fmr1*^−/y^ mice retinal molecular phenotype due to loss of Fmrp is present before EO with consequences on retinal function in young and adult mice. Our work suggests a critical role of the sensorial peripheral dysfunction in the *Fmr1*^−/y^ overall phenotype, and provides evidence that altered peripheral perception is a component of the sensory processing defect in FXS conditions. Thus, peripheral sensorial dys-sensitivity might lead to a misconception of the environment, and therefore might contribute to the exacerbation of the behavioral phenotype of FXS.

## Materials and Methods

### Animals

*Fmr1*^−/y^ males and their wild-type (WT) littermates were generated by breeding heterozygous *Fmr1*^+/–^ females with C57BL/6J background WT males. Mice were weaned at 21 days of age and group-housed with their same-sex littermates. On the same day, tail samples were collected for DNA extraction and for subsequent PCR assessment of genotypes as previously described (Bakker et al., 1994). Food and water were provided *ad libitum*. Animals were maintained under temperature (22°C) and humidity (55%) controlled conditions with a 12:12 h dim light–dark cycle (25 lux, lights on at 7 a.m.). All animal experimental protocols were reviewed by the “Ethics Committee for Animal Experimentation of CNRS Campus Orleans” (CCO N°3) and approved by the French National Committee of Ethical Reflexion for Animal Experimentation, under N° CLE CCO 1100.

### Experimental Design

*Fmr1^−/y^* mice male mice were investigated before and after the EO. The 1 day post-natal (1 DPN) time (day 1 postpartum), immediately after birth, was chosen since it covers the final stages of glial and neuronal proliferation and migration, axonal migration and synaptogenesis in the retina (Dorrell et al., 2004) without light experience. After EO, young (1 month-old) and adult (3 and 6 month-old) mice were selected since they fully express the *Fmr1*^−/y^ phenotypes (McNaughton et al., 2008; Hébert et al., 2014; Gauducheau et al., 2017). Therefore, different groups of animals were investigated: a first group of animals was used for functional (see “*Electroretinography—ERG*” section), histological (see “*Retinal*” section) and apoptotic cell (see “*TUNEL Assays*” section) analysis. Among these mice, one part was sacrificed at 1 month of age (WT *n* = 10; *Fmr1*^−/y^ *n* = 10). Remaining mice were sacrificed at 3 months of age (WT *n* = 10; *Fmr1*^−/y^ *n* = 10) and at 6 months of age (WT *n* = 10; *Fmr1*^−/y^ *n* = 10) after electroretinography. A second group of animals was used for *in vivo* investigation of retinal structures by OCT (see “*Optical Coherence Tomography—OCT*” section) at 3 months of age (WT *n* = 10; *Fmr1*^−/y^ *n* = 10). A third group of animals was used to evaluate the time pattern of retinal defects through molecular analysis by Western blot and qRT-PCR. They underwent molecular analysis (see “*Western-Blotting* and* Quantitative RT-PCR*” sections) before EO at 1DPN (WT *n* = 13; *Fmr1*^−/y^ *n* = 13), and at 1, 3 and 6 months of age (WT *n* = 13; *Fmr1*^−/y^ *n* = 13 for each age). Part of the 1 DPN sacrificed animals were also used for histological analysis (WT *n* = 2; *Fmr1*^−/y^ *n* = 2; see “*Retinal*” section).

### *In Vivo* Electroretinography (ERG)

After overnight dark adaptation, animals were anesthetized with ketamine (50 mg/kg) and xylazine (2 mg/kg). Eye drops were used to dilate the pupil (Atropine sulfate 1%, ALCON). Mice were placed on a temperature-regulated heating pad throughout the recording session. ERGs were recorded using two Ag/AgCl electrodes, one in contact with the corneal surface and one placed on the tongue. A copper reference screen under the animal was used as reference. Strobe flashs (10 μs) were presented through an integrating sphere (Labsphere, France) that mimics a Ganzfeld and allows to illuminate uniformly the whole retina. ERGs were recorded using increasing luminance from −3.47 to +0.46 log cd s/m^2^. Conversely, the duration of the interstimulus interval was 30 s since this interval had been shown to be sufficient for a flash not to alter the next flash response. Responses were differentially amplified (0.3–10,000 Hz), averaged, and stored. Intensity–response functions were obtained in a single session. At the end of the session, Oscillatory Potentials (OPs) are recorded by switching the amplifier to 100–300 Hz.

#### ERG Analysis

**Figure 1:**
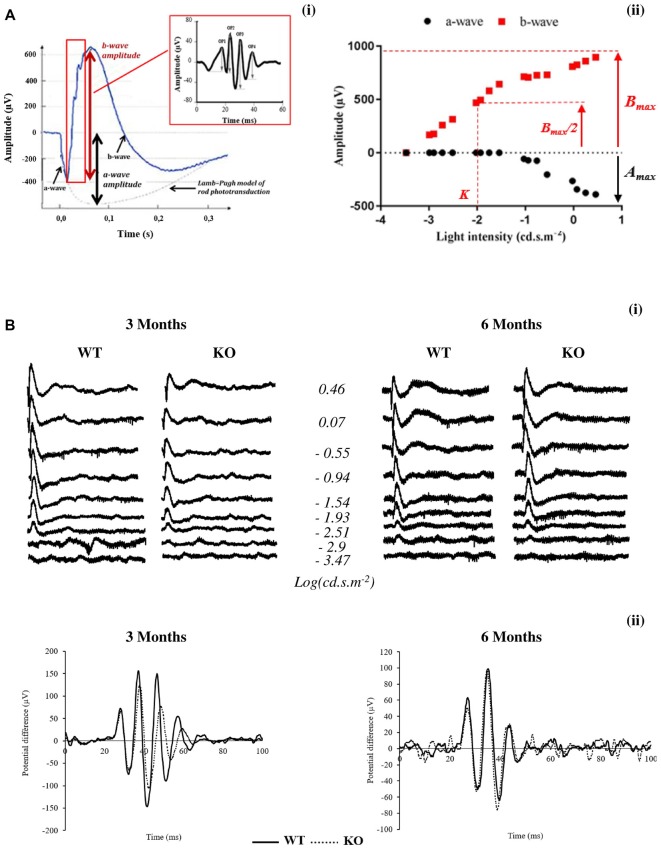
Scotopic electroretinogram (ERG) in wild-type (WT) and *Fmr1*^−/y^ (KO) mice at 3 and 6 months old.** (A)** Typical ERG for one flash stimulus with the **(i)** oscillatory potentials (OPs) in the b-wave ascending part and **(ii)** intensity–response function of a- and b-wave amplitudes are represented.** (B)** Representative **(i)** ERG and **(ii)** OPs traces obtained from WT or *Fmr1*^−/y^ mice at 3 and 6 months old.

Typically, an ERG (Figures 1Ai,Bi) is characterized by a negative deflection termed the a-wave, which is initiated by the activity of light-sensitive photoreceptor cells. The following positive deflection, termed the b-wave, reflects signal transmission to the inner retina, mainly due to light-induced activity of bipolar cells but also due to Müller cells involvement (Dong and Hare, 2000; Frischman, 2006). The small ripples on the ascending part of the b-wave, called OPs, involve multiple components, presumably including outer and inner retinal circuitry (Frischman, 2006).

The leading edge of the a-waves obtained in response to the highest-intensity stimuli (2.88 cd.s.m^−2^) was analyzed with a modified form of the Lamb–Pugh model of rod phototransduction (Granit, 1933; Lamb and Pugh, 1992) equation: P3 = {1−exp[i−S*_A_*(*t*−*t*_d_)^2^]}*A*_max_ where P3 represents the massed response of the rod photoreceptors and is analogous to the PIII component of Granit (Granit, 1933). The amplitude of P3 is expressed as a function of flash energy (*i*) and time (*t*) after flash onset. *S*_A_ is the gain of phototransduction, *A*_max_ is the maximum response, and *t*_d_ is a brief delay.

For each stimulus luminance, the b-wave amplitude was calculated from the minimum of the a-wave to the maximum of the b-wave. Intensity–response function of the b-wave amplitude (Figure 1Aii) was fitted with the Naka–Rushton equation: *B*/*B*_max_ = *I^n^*/(*I^n^*+*K^n^*) where *I* is the stimulus luminance of the flash, *B* is the b-wave amplitude of ERG at *I* luminance, *B*_max_ is the maximal b-wave amplitude, *K* is the half-saturation constant corresponding to retinal sensitivity and *n* is a dimensionless constant controlling the slope of the function.

*B*_max_/*A*_max_ ratio was calculated with *B*_max_ and *A*_max_ values obtained at the highest-intensity stimuli (2.88 cd.s.m^−2^).

OP (OP1 to OP4) amplitudes (Figure 1Bii) are calculated from the baseline to the maximum of the potential.

Latency is the time interval between the stimulation and the peak of the waves.

### Retinal Histology

Retinal histology was done as described previously (Chang et al., 2011; Rossignol et al., 2014). The retinal tissue sections were scanned for evidence of gross defects at 1DPN and at 1, 3 and 6 months old. For the other adult ages, retinal thickness of the total retina (Ret), Outer Nuclear Layer (ONL), Outer Plexiform Layer (OPL), Inner Nuclear Layer (INL) and Inner Plexiform Layer (IPL) were measured. In each retinal section, the measurement of thickness was made at 0.78 mm and 1.56 mm from the optic nerve to the inferior and to the superior *ora serrata*. All these results of measurements were then averaged per experimental groups. All measurements were performed with a Leica microscope (×40, Leica, Paris, France) and the ImageJ image processing program (National Institute of Health).

### Optical Coherence Tomography—OCT

Mice were anesthetized (Ketamine at 20 mg/ml and Xylazine at 1.17 162 mg/ml) and their pupils dilated with 10% phenylephrine and 0.5% tropicamide provided as eye drops (Systane Ultra, Alcon). OCT was performed for mouse retinas with the spectral domain (SD) ophthalmic imaging system as described previously (Jagodzinska et al., 2017). Thickness of retinal layers was measured manually with ImageJ image processing program (National Institute of Health) at 0.3 mm distance from the optic nerve.

### TUNEL Assays

TUNEL assays were conducted with an ApopTag^®^ Red *in situ* Apoptosis Detection Kit (S7165, EMD Millipore) following the indicated protocol. Briefly, sections were treated as indicated above, fixed in 1% PFA in PBS pH 7.4, washed in TBS pH 7.4 three times, incubated in equilibration pH buffer (potassium cacodylate; provided in the kit) for 10 min and incubated with terminal deoxynucleotidyl transferase for 60 min at 37°C. After 10 min in stop buffer (provided in the kit), sections were incubated with anti-digoxigenin conjugate overnight at 4°C. After washing in TBS pH 7.4, sections were counterstained with DAPI (10 μg/ml, Sigma), mounted in Fluoromount-G (SouthernBiotech) and examined with a fluorescence microscope (Leica, Paris, France). The number of apoptotic cells and number of total nuclei of ONL and INL layers were counted from the optic nerve to the superior left *ora serrata* and from the optic nerve to the superior right *ora serrata*. Light-exposed (24 h, 3500 lux) albinos retinas (3 months old) were used as control (Ctrl, Figure 3C; Perche et al., 2009).

**Figure 2:**
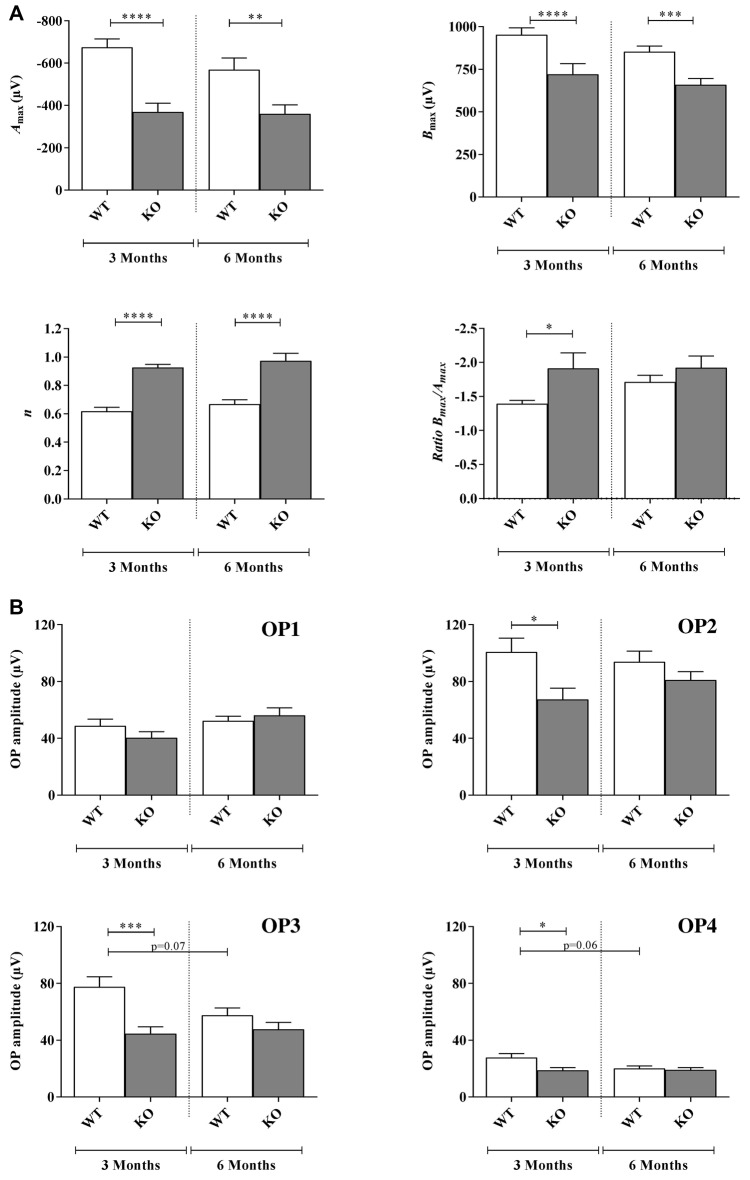
Scotopic ERG parameters measured in WT and *Fmr1*^−/y^ (KO) mice at 3 and 6 months old. **(A)** Retinal function was assessed by recording ERG (WT *n* = 10 and *Fmr1*^−/y^ *n* = 10 for each age). For each typical ERG obtained at light intensity −2.88 log(cd.s.m^−2^), the decreasing part of the a-wave was fitted to calculate the extrapolated maximal a-wave amplitude (*A*_max_). From the fitted b-wave sensitivity curve obtained by serial responses to increasing flash stimuli (−3.47 log(cd.s.m^−2^) to 0.6 log(cd.s.m^−2^)) we calculated the saturated b-wave amplitude (*B*_max_) and the *n* parameter (representing the b-wave sensitivity curves slope). Ratio *B*_max_/*A*_max_ was also calculated. **(B)** OPs result in the ascending part of the b-wave. OPs were recorded by using a band-pass between 30 Hz and 300 Hz. For each OPs, the amplitude from the baseline to the peak and the latency were calculated. Data are presented as Mean ± SEM. Significant differences between WT and *Fmr1*^−/y^ for one age time are noted by *p < 0.05; **p < 0.01; ***p < 0.001; ****p < 0.0001.

**Figure 3:**
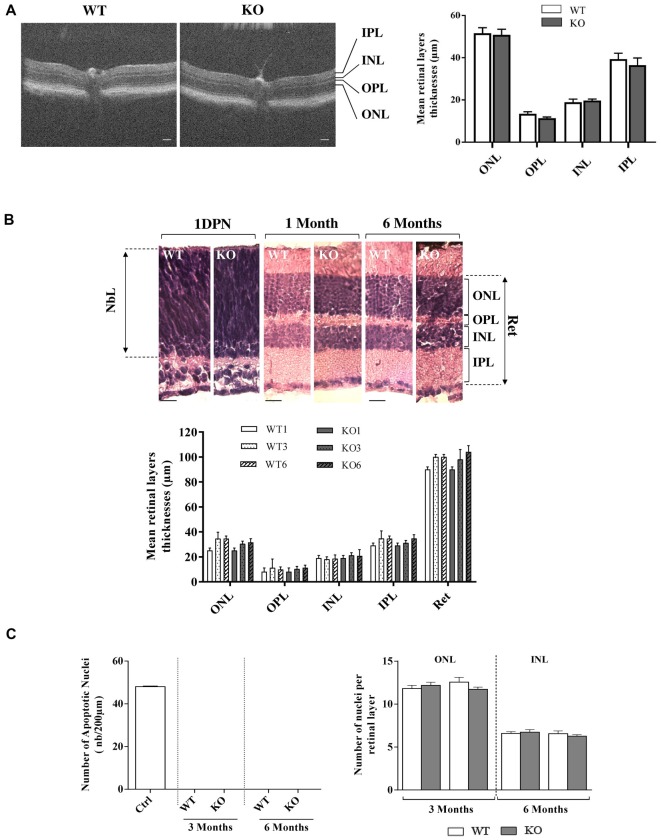
Retinal histology and TUNEL assay in WT and *Fmr1*^−/y^ (KO) mice. **(A)** Retinal layer structure was evaluated *in vivo* by OCT at 3 months old (WT *n* = 10 and *Fmr1*^−/y^ *n* = 10; Scale bar, 50 μm) and **(B)** by histology techniques at 1 day post-natal (1DPN; WT *n* = 2 and *Fmr1*^−/y^ *n* = 2) and at 1, 3 and 6 months old (noted WT1 and KO1, WT3 and KO3 and WT6 and KO6 respectively, WT *n* = 10 and *Fmr1*^−/y^ *n* = 10 for each age; Scale bar, 20 μm; **C**) On the 3 and 6 months old sections, number of apoptotic cells per mm^2^, assessed by TUNEL assay, and total number of nuclei in the ONL and INL was counted (WT *n* = 5 and *Fmr1*^−/y^ *n* = 5 for each age). Data are presented as Mean ± SEM (NbL, Neuroblastic Layer; ONL, Outer Nuclear Layer; OPL, Outer Plexiform Layer; INL, Inner Nuclear Layer; IPL, Inner Plexiform Layer or Ret: Total Retina).

### Western Blotting

Proteins were extracted from mouse whole retinas as described previously (Rossignol et al., 2014). Briefly, the two retinas from the same mouse were homogenized in RIPA buffer (10 mM Tris-HCl, pH 7.6; 1 mM EDTA; 0.15 mM NaCl; 1% Igepal; 0.2% SDS) supplemented with protease cocktail inhibitors (Pierce, Paris, France). Protein concentration was determined in the supernatant by BCA protein assay kit (Pierce, Paris, France). Protein samples (15 μg) were run on SDS/PAGE gels (8%–12%, w/v), transferred to a nitrocellulose membrane, and probed with primary antibodies (anti-Fmrp, 1:1000, anti-Rhodopsin, 1:200, anti-PSD95, 1:500) and secondary HRP-antibody (1:4000) purchased from AbCam (Paris, France), Sigma-Aldrich (Illkirch, France) and Phosphosolutions (Cliniscience, France). On the same blots, protein contents were normalized to the amounts of β-actin (mouse anti-beta Actin antibody; 1:4000; AbCam, Paris, France). Quantification of immunoreactive bands was done using Western Blotting Imager PXi4 (Ozyme, Paris, France). Mean values in each group were expressed as percentage of WT mice. Western-blots were performed three times. Representative results are shown in figures.

### Quantitative RT-PCR

Quantitative RT-PCR was performed using Taqman technologies (Applied technologies) as described previously (Rossignol et al., 2014). Briefly, total RNA was isolated from WT and *Fmr1*^−/y^ retinas using Trizol reagent (Ambion, Life Technologies), quantified and reverse transcripted (Superscript iii reverse transcriptase, Invitrogen, Life Technologies). Real-time PCR reactions were performed in the Mx3005P Agilent (Applied, Life Technologies) with fivefold dilution of cDNA, 200 nM of each Taqman primer using the Expression Master Mix (Applied, Life Technologies). Data were analyzed by ΔΔCt method and normalized to the reference standard RNA 18S. Each measurement was performed three times.

### Statistical Analysis

All results are expressed as mean ± SEM. Data analysis was performed using GraphPad Prism 7.00. Statistical analysis was conducted using two-Way ANOVA with age (*F*_a_) and genotype (*F*_g_) as main factors followed by *post hoc* Tukey’s HSD-test when a statistically significant main effect or interaction was detected (*p* < 0.05). For OCT measurements, statistical analysis was conducted using one-Way ANOVA followed by *post hoc* Tukey’s HSD. Statistical significance was defined as *p* < 0.05. In figures, significant differences between genotypes are noted by *p < 0.05; **p < 0.01; ***p < 0.001; ****p < 0.0001, and difference between ages are noted ^$^*p* < 0.05; ^$$^*p* < 0.01; ^$$$^*p* < 0.001; ^$$$$^*p* < 0.0001.

## Results

### Major Retinal Function Alteration in *Fmr1*^−/y^ Without Retinal Structural Modification

In order to better understand the retinal function of the *Fmr1*^−/y^ mice, we recorded the electrophysiological response of the retina to light stimulation, called ERG (Figures 1Ai,Bi). For ERG parameters see “*In Vivo Electroretinography*” section in “Materials and Methods” paragraph. ERGs were recorded at 3 and 6 months of age.

#### A-Wave Investigation

For each ERG recorded at the highest light stimulus, the decreasing part of the a-wave was fitted to calculate the maximal a-wave amplitude (*A*_max_), the parameter S*A* reflecting photoreceptor sensitivity and the a-wave latency corresponding to the time between light stimulation and *A*_max_ maximal response.

Regarding the *A*_max_ parameter, the photoreceptor response, no significant interaction between genotype and age were noticed (*F*_a,g(1,76)_ = 2.029, *p* = 0.1584). Moreover, interaction remained stable between 3 and 6 months old in WT (−674 ± 59 μV and −568 ± 74 μV, respectively) and *Fmr1*^−/y^ mice (−368 ± 60 μV and −359 ± 55 μV, respectively; *F*_a(1,76)_ = 2.792, *p* = 0.988; Figure 2A). However, *Fmr1*^−/y^ retinas had a significantly lower (*F*_g (1,76)_ = 43.59, *p* < 0.0001) photoreceptor response than WT (*post hoc*: *p* < 0.0001 and *p* = 0.0026, respectively). There was no significant variation in a-wave latency irrespectively of age or genotype (data not shown).

No significant interaction between genotype and age was noticed (*F*_a,g(1,71)_ = 0.01687, *p* = 0.8970) for the parameter *S*_A_ reflecting photoreceptor sensitivity. Moreover, interaction was similar between 3 months and 6 months old mice in WT (0.0012 ± 0.0001 μV and 0.0012 ± 0.0001 μV, respectively) or *Fmr1*^−/y^ mice (0.0016 ± 0.0002 μV and 0.0016 ± 0.0002 μV, respectively; *F*_a(1,71)_ = 0.0015, *p* = 9689). In addition, there was no significant variation between *Fmr1*^−/y^ and WT mice (*F*_g (1,71)_ = 2.45, *p* = 0.1218). Therefore, *Fmr1*^−/y^ retinas did not present alteration of photoreceptor sensitivity to light.

#### B-Wave Investigation

B-wave amplitude was plotted as a function of stimulus luminance to obtain a b-wave sensitivity curve (Figure 1Aii). For each animal, the b-wave sensitivity curve was fitted to calculate the maximal b-wave amplitude (*B*_max_) reflecting the maximal retinal response, the half saturation luminance (*K*) reflecting the light intensity generating half *B*_max_, and the slope of the curve in its linear part (*n*) reflecting the contrast sensitivity of retina.

Regarding the* B*_max_ no significant interaction between genotype and age was noticed (*F*_a,g(1,76)_ = 0.2797, *p* = 0.5984) since it remained stable between 3 months and 6 months old WT (952 ± 40 μV and 853 ± 33 μV, respectively) and *Fmr1*^−/y^ (720 ± 62 μV and 659 ± 35 μV, respectively) mice (*F*_a(1,76)_ = 2.783, *p* = 0.0994; Figure 2A). However, *Fmr1*^−/y^ retinas had a significantly lower maximal b-wave amplitude at 3 and 6 months of age (*F*_g (1,76)_ = 43.46, *p* < 0.0001; *post hoc*: *p* < 0.0001 and *p* = 0.0003, respectively) compared to WT ones. In addition, irrespectively of age or phenotype b-wave latency was similar (data not shown).

The half saturation luminance (*K*) was not significantly different between 3 months and 6 months old WT (−1.93 ± 0.11 and −2.10 ± 0.05, respectively) or *Fmr1*^−/y^ (−2.03 ± 0.17 and −2.01 ± 0.07) mice (*F*_ag (1,76)_ = 2.537, *p* = 0.11.54; *F*_a(1,76)_ = 2.286, *p* = 0.5941). Differences between WT and *Fmr1*^−/y^ mice were not significant (*F*_g (1,76)_ = 0.006, *p* = 0.9372).

The *n* parameter did not significantly change between 3 months and 6 months old WT or *Fmr1*^−/y^ mice (*F*_ag (1,68)_ = 0.002, *p* = 0.9614; *F*_a(1,76)_ = 1.845, *p* = 0.1789; Figure 2A). However, *Fmr1*^−/y^ mice values were significantly higher (*F*_g (1,76)_ = 51.08, *p* < 0.0001) than WT ones at both 3 (0.62 ± 0.02 WT vs. 0.92 ± 0.02 *Fmr1*^−/y^, *post hoc*: *p* < 0.0001) and 6 months old (0.66 ± 0.02 WT vs. 0.97 ± 0.05 *Fmr1*^−/y^, *post hoc*: *p* < 0.0001). Therefore, *Fmr1*^−/y^ retinas showed a lower contrast sensitivity compared to WT ones.

#### *B*_max_/*A*_max_ Ratio Investigation

The *B_max_/A_max_* ratio slightly increased between 3 months and 6 months old in WT retinas (−1.39 ± 0.05 and −1.71 ± 0.09, respectively) but remained stable in *Fmr1*^−/y^ retinas (−1.91 ± 0.22 and −1.92 ± 0.17, respectively; *F*_ag (1,73)_ = 2.31, *p* = 0.1329; *F*_a(1,73)_ = 0.4207, *p* = 5186; Figure 2A). However, *Fmr1*^−/y^ retinas had a significantly lower ratio at 3 months old compared to WT (*F*_g (1,73)_ = 5.909, *p* = 0.0175;* post hoc*: *p* = 0.0382), but not anymore at 6 months (*post hoc*: *p* = 0.923) due to the WT increase.

#### Oscillatory Potentials Investigation

Amplitude of the small ripples on ascending part of the b-wave, called OPs (Figure 1Ai), are represented for each age and genotype in Figure 2B. For each OP, no significant interaction between genotype and age was noticed (OP1: *F*_a,g(1,74)_ = 0.3539, *p* = 0.5537; OP2: *F*_a,g(1,74)_ = 0.1382, *p* = 0.2431; OP3: *F*_a,g(1,82)_ = 3.514, *p* = 0.0644; OP4: *F*_a,g(1,80)_ = 2.814, *p* = 0.0974). Moreover, no effect of age was observed (OP1: *F*_a(1,82)_ = 3.638, *p* = 0.0603; OP2: *F*_a(1,82)_ = 0.1451, *p* = 0.7043; OP3: *F*_a(1,823)_ = 1.865, *p* = 0.1758; OP4: *F*_a(1,80)_ = 0.2.470 *p* = 0.1200) while a main genotype effect was noticed for OP2, OP3 and OP4 (OP2: *F*_g(1,74)_ = 6.890, *p* = 0.0103; OP3: *F*_g (1,82)_ = 11.99, *p* = 0.0009; OP4: *F*_g (1,80)_ = 4.536, *p* = 0.0363) but not for OP1 (OP1: *F*_g (1,74)_ = 3.638, *p* = 0.0603). In WT, OP1 and OP2 amplitudes remained stable between 3 months and 6 months of age (OP1: from 48 ± 4 μV to 52 ± 3 μV; OP2: from 100 ± 9 μV to 93 ± 7 μV; Figure 2B). However, OP3 and OP4 amplitudes tended to decrease between 3 months and 6 months but the difference was not significant (OP3: from 77 ± 7 μV to 57 ± 5 μV, *post hoc*: *p* = 0.0785; OP4: from 27 ± 3 μV to 20 ± 2 μV, *post hoc*: *p* = 0.067; Figure 2B). In *Fmr1*^−/y^ mice, OP1, OP2, OP3 and OP4 did not significantly vary between 3 months and 6 months (OP1: from 40 ± 4 μV to 56 ± 5 μV, *p* = 0.039; OP2: from 67 ± 7 μV to 80 ± 5 μV; OP3: from 44 ± 4 μV to 47 ± 4 μV and OP4: from 19 ± 2 μV to 19 ± 2 μV; Figure 2B). However, OP2, OP3 and OP4 were significantly decreased in *Fmr1*^−/y^ retinas compared to WT ones at 3 months of age (*post hoc*: OP2: *p* = 0.022 ; OP3: *p* = 0.0005; OP4: *p* = 0.018) but not anymore at 6 months. The loss of difference is explained by the decrease observed in WT values while *Fmr1*^−/y^ ones remained stable between 3 months and 6 months of age. OP latencies were not different between WT and *Fmr1*^−/y^ mice whatever the age (data not shown).

#### Retinal Histology

We first investigated *in vivo* retinal structure by OCT technique, measuring layer thickness at 3 months (Figure 3A). Whatever was the considered layer (ONL, OPL, INL, IPL), no significant difference in thickness was observed between WT and *Fmr1*^−/y^ retinas (ONL: *p* = 0.840; OPL: *p* = 0.111, INL: *p* = 0.630; IPL: *p* = 0.531). Then we investigated gross retinal histology throughout ages in WT and *Fmr1*^−/y^ retinas by measuring retinal layer thicknesses (Figure 3B). These thicknesses were not different between WT and *Fmr1*^−/y^ mice whatever was the age or the considered layer (ONL: *F*_a,g(2,54)_ = 0.279, *p* = 0.757; *F*_a(2,54)_ = 1.205, *p* = 0.307; *F*_g (2,54)_ = 1.066, *p* = 0.306–OPL: *F*_a,g(2,54)_ = 0.050, *p* = 0.951; *F*_a(2,54)_ = 0.3621, *p* = 0.697; *F*_g (2,54)_ = 0.002, *p* = 0.962–INL: *F*_a,g(2,54)_ = 0.153, *p* = 0.857; *F*_a(2,54)_ = 0.033, *p* = 0.967; *F*_g (2,54)_ = 0.561, *p* = 0.457–IPL: *F*_a,g(2,54)_ = 0.224, *p* = 0.799; *F*_a(2,54)_ = 1.671, *p* = 0.197; *F*_g (2,54)_ = 0.210, *p* = 0.648–Total Retina (Ret): *F*_a,g(2,54)_ = 0.409, *p* = 0.666; *F*_a(2,54)_ = 0.866, *p* = 0.426; *F*_g (2,54)_ = 0.009, *p* = 0.922). This is consistent with *in vivo* observation. Interestingly, we found that even at the neonatal stage (1DPN) meaning before appearance of ONL and INL from the neuroblastic layer (NbL; Yu et al., 2011), there were no obvious differences in the overall retina structures between WT and *Fmr1*^−/y^ (Figure 3B). These histological data were reinforced by TUNEL) staining showing no apoptotic retinal cells and no significant difference of total nuclei number in ONL (*F*_a,g(1,55)_ = 2.564, *p* = 0.115; *F*_a(1,55)_ = 0.155, *p* = 0.695; *F*_g(1,55)_ = 0.321, *p* = 0.573) and INL (*F*_a,g(1,55)_ = 0.764, *p* = 0.385; *F*_a(1,55)_ = 0.976, *p* = 0.327; *F*_g(1,55)_ = 0.117, *p* = 0.733) layers (Figure 3C).

Therefore, ERG parameters showed altered retinal function in *Fmr1*^−/y^ compared to WT mice, without any gross retinal structure modifications.

### Early Molecular Impairments in *Fmr1*^−/y^ Retinas

**Figure 4:**
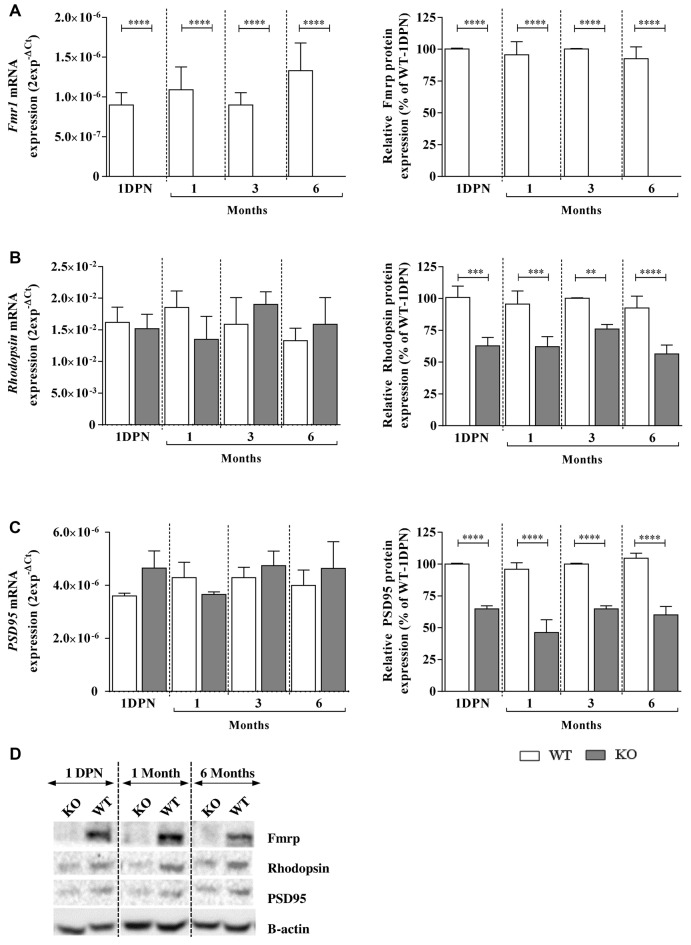
Fragile X mental retardation protein (Fmrp), Rhodopsin and PSD95 mRNA and protein expressions in WT and *Fmr1*^−/y^ (KO) mice at 1 DPN and 1, 3 and 6 months old. mRNA and protein expressions of **(A)** Fmrp, **(B)** Rhodopsin and **(C)** PSD95 were assessed by qPCR (*n* = 8 per group) and Western-blot (*n* = 5 per group) analysis in WT (white bars) and *Fmr1*^−/y^ (gray bars) mice. For qPCR, data are expressed as 2^−ΔCt^ values and normalized to 18S RNA internal control. For Western-blot, data are presented as Mean ± SEM in percentage of WT aged of 1 DPN. **(D)** A representative Western-blot experiment obtained for WT and *Fmr1*^−/y^ (KO) mice is presented for each protein at 1 DPN, 1 and 6 months old. Significant differences between groups are noted by **p < 0.01; ***p < 0.001; ****p < 0.0001.

In the retina, Fmrp was expressed in WT mice from 1 DPN, before EO, to 6 months old without any variation of protein nor mRNA (Figure 4A) expression (*F*_a,g(3,119)_ = 2.076, *p* = 0.1071, *F*_a,g(3,128)_ = 0.3717, *p* = 0.7736; *F*_a(3,119)_ = 2.076, *p* = 0.1071, *F*_a(3,128)_ = 0.4894, *p* = 0.6902, respectively). In *Fmr1*^−/y^ mice, Fmrp was always absent (*F*_g (1,119)_ = 263.9, *p* < 0.0001, *F*_g (1,128)_ = 1423, *p* < 0.0001, respectively; Figures 4A,D).

Regarding Rhodopsin and PSD95 mRNA investigations, no age (*F*_a,g(3,119)_ = 0.2923, *p* = 0.8309, *F*_a,g(3,128)_ = 1.416, *p* = 0.2415; *F*_a(3,119)_ = 0.1392, *p* = 0.9363; *F*_a(3,119)_ = 0.4784, *p* = 0.6979, respectively) or genotype (*F*_g (1,119)_ = 0.05769, *p* = 0.8106; *F*_g (1,119)_ = 1.577, *p* = 0.2116, respectively) effects were noticed. However, the protein study showed no age effect (*F*_a,g(3,115)_ = 0.4508, *p* = 0.7172, *F*_a,g(3,117)_ = 2.262, *p* = 0.0849; *F*_a(3,115)_ = 2.609, *p* = 0.549; *F*_a(3,117)_ = 1.995, *p* = 0.1185, respectively) but a main genotype effect (*F*_g (1,115)_ = 85.63, *p* < 0.0001; *F*_a(1,117)_ = 701.9, *p* < 0.0001, respectively). Thus, in WT retinas, Rhodopsin mRNA and protein were detected and their contents were similar before EO and at young or adult ages (Figures 4B,D). In *Fmr1*^−/y^ retinas, Rhodopsin mRNA expression was similar from 1 DPN to 6 months old, and similar to the one observed in WT retinas (Figure 4B). However, there was a significant decrease in Rhodopsin protein content by about 30%–40% in *Fmr1*^−/y^ compared to WT at 1 DPN (100 ± 8% in WT vs. 62 ± 6% in *Fmr1*^−/y^, *post hoc*: *p* = 0.0001), at 1 month (99 ± 10% in WT vs. 62 ± 8% in *Fmr1*^−/y^*, post hoc*: *p* = 0.0005), at 3 months (100 ± 1% in WT vs. 70 ± 3% in Fmr1^−/y^, *post hoc*: *p* = 0.0033) and at 6 months (93 ± 9% in WT vs. 56 ± 7% in *Fmr1*^−/y^, *post hoc*: *p* < 0.0001; Figures 4B,D). In WT retinas, PSD95 mRNA and protein contents (Figure 4C) were similar before EO and at young or adult ages. In *Fmr1*^−/y^ retinas, PSD95 mRNA expression was similar from 1 DPN to 6 months old, and similar to the one observed in WT retinas (Figure 4C). However, there was a significant decrease in PSD95 protein content by about 30%–40% in *Fmr1*^−/y^ compared to WT at 1 DPN (100 ± 1% WT vs. 64 ± 2% *Fmr1*^−/y^, *post hoc*: *p* < 0.0001), at 1 (96 ± 5% WT vs. 46 ± 10% *Fmr1*^−/y^, *post hoc*: *p* < 0.0001), 3 (100 ± 1% WT vs. 64 ± 2% *Fmr1*^−/y^, *post hoc*: *p* < 0.0001) and 6 (104 ± 4% WT vs. 60 ± 6% *Fmr1*^−/y^, *post hoc*: *p* < 0.0001) months old compared to WT one (Figures 4C,D).

Therefore, even before EO until adult age, *Fmr1*^−/y^ retinas presented Rhodopsin and PSD95 protein defects without alteration of their mRNA expression.

## Discussion

In the absence of FMRP, a RNA-binding protein involved in protein translation, several proteic dysregulations (Darnell et al., 2005, 2011) had been shown to lead to cerebral neuronal immaturity and synaptic defects (Irwin et al., 2000; Nimchinsky et al., 2001). These cellular impairments responsible for functional and behavioral phenotypes of FXS (Penagarikano et al., 2007; Hagerman and Hagerman, 2015), are also noticed in *Fmr1*^−/y^ mice, the murine model of FXS (Hébert et al., 2014). Interestingly, our previous studies demonstrated that retina of this murine model presents molecular and cellular defects, similar to cerebral ones, in adult mice (Rossignol et al., 2014). However, no data are currently available on the specific time course of these abnormalities.

### Early Molecular Retinal Defects Are Leading to Lifetime Retinal Function Impairment With No Structural Impact

At a cerebral level, in FXS conditions (Irwin et al., 2000) as in the animal model (Irwin et al., 2000; Nimchinsky et al., 2001; Bilousova et al., 2009), FMRP deficiency leads to the neuronal immaturity phenotype caused by proteomic defects. Indeed, critical imbalance in translational mechanisms had been pointed out between WT and *Fmr1*^−/y^ mice during the cerebral synaptogenesis stage and adulthood (Zhu et al., 2011; Tang et al., 2015). Among the wide panel of misregulated proteins, a major part is composed of scaffold proteins for the synaptic structure present at pre- and post-synaptic levels, such as Shank1, Shank2 and PSD95. As in the brain, synaptic protein defects are also observed in the *Fmr1*^−/y^ retina. Indeed, we showed a reduced level of the synaptic scaffold protein PSD95 from 1 DPN up to 6 months without mRNA alteration. This data also confirmed that the absence of Fmrp in the retina affects the translation and not the mRNA transcription (Darnell et al., 2005, 2011). In addition, we assume that other proteins could participate (contribute) to this early retinal immaturity phenotype in synergy with PSD95 since other synaptic proteins, such as Syt1a, had been shown deregulated in the *Fmr1*^−/y^ retinas (Rossignol et al., 2014). Interestingly, all these impairments are associated, at least in adulthood, with retinal neuronal immaturity (Rossignol et al., 2014) without gross retinal structural modifications.

Early *Fmr1*^−/y^ retinal defects in protein expression lead coherently to abnormalities in retinal function in young and adult mice, as observed by ERG. ERG is defined as the specific response of the retina to light and represents the electrophysiological manifestations from Rhodopsin activation by light into electrophysiological message sent through the optic nerve to the brain (Fox and Rubinstein, 1989; Fulton et al., 1999). Our study highlighted similar alterations at all tested ages. The drop of a-wave amplitude is consistent with the decrease of Rhodopsin expression and reflects a reduced activation of photoreceptor cells in response to a light stimulation. Indeed, the direct relationship between Rhodopsin content and a-wave amplitude had been previously described (Liang et al., 2004; Price et al., 2012). The photoreceptor less activated, the signal transmitted to the inner retina is reduced leading to a decrease of the b-wave amplitude. However, the increased *B*_max_/*A*_max_ amplitude ratio in *Fmr1*^−/y^ compared to WT mice suggests a higher signal amplification in the transmission between photoreceptors and the inner retina. These results suggest an over activation in response to a given light intensity, from an electrophysiological point of view. This alteration is associated with a lower contrast sensitivity as shown by the increase in the parameter *n* (Ranchon et al., 1999). Moreover, retinal inner cells are also impacted in *Fmr1*^−/y^ mice since OPs, reflecting spatial and temporal integration of the retinal information by bipolar cells (Wachtmeister, 1998; Akula et al., 2007) are decreased. It reinforced our previous observation on synaptic defect in *Fmr1*^−/y^ (Rossignol et al., 2014). In summary, in *Fmr1*^−/y^ our results demonstrate a collapse in the capacity of retinal neurons to relay correctly visual signal, from youth with no evolution to adulthood. Consequently, we assume that the misperception of contrast, texture and moving stimuli described as the consequence of visual cerebral integration defects in FXS patients (Kogan et al., 2008; Farzin et al., 2011) must have a significant retinal component, as suggested by our retinal function data.

The early molecular retinal phenotype of the *Fmr1*^−/y^ mice is a major result of our work since it occurs before the EO. Indeed, EO is a crucial step for cerebral cortical maturation (Gandhi et al., 2008) since the primary visual cortex undergoes considerable synaptogenesis after EO (Blue and Parnavelas, 1983; Gandhi et al., 2005). This retinal signal leads to central visual synapses maturation through the redistribution of PSD95 in cerebral dendrites (Yoshii et al., 2003). Thus, induction of cerebral visual system maturation is essentially due to the retinal signal, resulting from retinal light sensing due to Rhodopsin, rods photopigment, retinal neuronal connections partially due to the synaptic protein PSD95 and also the retinal ganglion cells spontaneous activity (Blue and Parnavelas, 1983; Tian, 2004; Gandhi et al., 2005). In *Fmr1*^−/y^ retinas, we found that PSD95 and Rhodopsin proteins are depleted at 1 DPN, so even before EO. Since these protein defects are similar before EO and at adulthood with, at least in adult ages, a retinal neuronal immaturity (Rossignol et al., 2014), it seems straightforward to hypothesize that retinal function alterations observed at adulthood are also present even before EO. Therefore, when EO occurs, retinal light sensing is already altered. It becomes obvious that in *Fmr1*^−/y^ mice, the retinal signal for cerebral maturation is damaged. Since loss of Fmrp leads to PSD95 alteration in *Fmr1*^−/y^ brains (Zhu et al., 2011) and to PSD95 and Rhodopsin defects in *Fmr1*^−/y^ retinas, we assume that the cerebral visual phenotype is the synergistic consequence of both retinal and cerebral alterations. Our results are consistent with the defects in spatiotemporal visual processing in FXS patients due to primary visual cortex immaturity (Kogan et al., 2008; Farzin et al., 2011; Berman et al., 2012). Therefore, we assume that the altered retinal perception of light stimuli is critically involved in the whole visual sensorial FXS phenotype.

### FXS Sensorial Phenotype: Hypersensitivity or Dys-Sensitivity?

Early in life, FXS patients present auditory, olfactory, nociceptive and visual abnormalities (Casamassimo et al., 1986; Lachiewicz et al., 1994), creating a wide range of phenotypical dysregulations in sensory responses. These sensorial perturbations may participate in occurrence of major behavioral troubles in FXS as suggested recently (Carreno-Munoz et al., 2018). According to our hypothesis, the term “sensorial hypersensitivity”, literally suggesting a higher sensorial response, should be avoided. Indeed, hypersensitivity phenotypes have been demonstrated for audition aspects as well as for visual responses since *Fmr1*^−/y^ mice present hyper arousal excitability for auditory processing and exacerbated transmission between photoreceptors and the inner retina (as observed in our experiment), respectively. But tactile nociception after local acute inflammation is lowered (Price et al., 2007; Busquets-Garcia et al., 2013), and *Fmr1*^−/y^ mice present a significant decrease in odorant sensitivity (Schilit Nitenson et al., 2015). Consequently, we propose that the most relevant terminology to characterize this complex sensorial spectrum is “dys-sensitivity phenotype” instead of “hyper-sensitivity phenotype”. To go further, we assume that peripheral as well as central components of senses should be investigated in sensorial sensitivity. Based on our results on vision, we clearly highlighted that the retinal altered response is involved in the overall visual defect of *Fmr1*^−/y^ mice. We have demonstrated for the first time that the retinal phenotype of *Fmr1*^−/y^ mice is an early and stable phenotype characterized by a global lower visual performance.

Finally, the wide panel of sensorial troubles in audition, olfaction, nociceptive response and vision leads probably to a misunderstanding of the outside environment by FXS patients. Therefore, we assume that the entire neurosensorial system, from the stimulus perception to stimulus integration, is altered and critically involved in the overall FXS phenotype. The current challenge may be to discriminate between peripheral and central components on the cognitive and behavioral phenotypes of FXS.

## Conclusion

FXS patients present global sensorial abnormalities, which were, up to now, associated to cerebral neuronal immaturity. Our study based on the *Fmr1*^−/y^ mice (murine model of FXS) demonstrates that retina, the visual perception tissue, presents early molecular and electrophysiological defects in young and adult mice. The molecular defects are settled even before EO, the decisive signal triggering for the central visual area maturation. Thus, our work on vision provides evidence that altered peripheral perception is a crucial component of the sensory processing defects of *Fmr1*^−/y^ mice. This peripheral dys-sensitivity is as important as the central sensorial defect in the FXS pathology.

## Author Contributions

OP, IR-C and CF: designed research study. OP, IR-C, MA, A-LM-B and CF: data analysis. OP, CF, AP, AB, RR, GM-D, A-LM-B and CM-D: conducted experiments. OP, CF, AP, AB, RR, GM-D, J-CB, CM-D, DL, BH, AM, JP and SB: discussed data. OP, CF, MA and SB: writing manuscript.

## Conflict of Interest Statement

J-CB was employed by company KeyObs. The other authors declare that the research was conducted in the absence of any commercial or financial relationships that could be construed as a potential conflict of interest.
